# Systems biology and metabolic engineering of *Arthrospira* cell factories

**DOI:** 10.5936/csbj.201210015

**Published:** 2012-12-09

**Authors:** Amornpan Klanchui, Tayvich Vorapreeda, Wanwipa Vongsangnak, Chiraphan Khannapho, Supapon Cheevadhanarak, Asawin Meechai

**Affiliations:** a Biochemical Engineering and Pilot Plant Research and Development Unit, National Center for Genetic Engineering and Biotechnology at King Mongkut's University of Technology Thonburi, Bangkhuntien, Bangkok, Thailand; b Microarray Laboratory, National Center for Genetic Engineering and Biotechnology (BIOTEC), KlongLuang, Pathumthani, Thailand; c Center for Systems Biology, Soochow University, Suzhou, Jiangsu 215006, China; d Devision of Biotechnology, School of Bioresources and Technology, King Mongkut's University of Technology Thonburi, Bangkok, Thailand; e Department of Chemical Engineering, Faculty of Engineering, King Mongkut's University of Technology Thonburi, Bangkok, Thailand

## Abstract

*Arthrospira* are attractive candidates to serve as cell factories for production of many valuable compounds useful for food, feed, fuel and pharmaceutical industries. In connection with the development of sustainable bioprocessing, it is a challenge to design and develop efficient *Arthrospira* cell factories which can certify effective conversion from the raw materials (i.e. CO_2_ and sun light) into desired products. With the current availability of the genome sequences and metabolic models of *Arthrospira*, the development of *Arthrospira* factories can now be accelerated by means of systems biology and the metabolic engineering approach. Here, we review recent research involving the use of *Arthrospira* cell factories for industrial applications, as well as the exploitation of systems biology and the metabolic engineering approach for studying *Arthrospira*. The current status of genomics and proteomics through the development of the genome-scale metabolic model of *Arthrospira*, as well as the use of mathematical modeling to simulate the phenotypes resulting from the different metabolic engineering strategies are discussed. At the end, the perspective and future direction on *Arthrospira* cell factories for industrial biotechnology are presented.

## 1. Introduction

The term “systems biology” appears widely in the literature and is defined as an emerging approach applied to biomedical and biological scientific research [1]. Systems biology mostly focuses on integrative omics data analysis, mathematical modeling, cellular components interactions, and quantification of dynamic responses in living organisms. Systems biology is typically proposed to achieve a quantitative biological system under study and this is often shown in the form of a mathematical model. A number of case studies use the model for capturing reporter features of the biological system and can hence be later used to predict cellular behaviors at different conditions [2]. In other cases, the modeling of the mathematical model rather serves as a toolbox to extract information of the biological system and to perform data enrichment and classification. Generally, this mathematical model goes hand in hand with laboratory experimental work. Through this, the essence combination exemplifies the core of systems biology and leads to gaining new insight into the fundamental molecular mechanisms occurring in living cells.

In recent years, systems biology has moved towards metabolic engineering [3]. Metabolic engineering is a fascinating science which has several definitions. Most of these are mostly aimed at improving existing cell factories or developing new ones, which are similar to the use of genetic engineering to perform directed genetic manipulations of cell factories with the overall objective to improve their properties for industrial applications. Nonetheless, metabolic engineering clearly differentiates itself by the use of advanced analytical techniques for the identification of suitable targets for genetic manipulation and the use of mathematical models to perform *in silico* design of optimized cell factories.

Focusing in the field of industrial biotechnology, there is much studied on how systems biology and metabolic engineering can impact the development of efficient cell factories, especially moving forward in improving industrial production processes and developing new products. In this present era, there are certainly rapid jumps towards the use of another type of microalgae, the cyanobacteria *Arthrospira* (formerly known as *Spirulina*), as sustainable cell factories. These bacteria are well-known to be used in the production of many industrial products, including high value compounds, such as healthy food supplements, animal feed, cosmetics and pharmaceutical products. In addition, *Arthrospira* are currently used as important green cell factories for biofuel production (e.g. hydrogen, bioethanol and biodiesel) which serve for renewable energy resources [4, 5].

With the advancement of high-throughput omics technologies and bioinformatics accompanied by systems biology and metabolic engineering, these processes have rapidly allowed for the obtainment of a more comprehensive understanding of *Arthrospira* cell factories. The basic researches of high-throughput omics technologies are generally focused on how metabolisms are operating at different environmental conditions. Therefore, integrative technologies like genomics, transcriptomics, proteomics, metabolomics and fluxomics as well as bioinformatics, which provide qualitative and/or quantitative information on the operation of the metabolism in a context of network, are playing a key role in systems biology. Besides these technologies, systematic techniques underlying metabolic engineering for the analysis of metabolic pathways and guiding rational strategies for the direct improvement of cellular activity [6, 7] are additionally valuable. Considering all possible advanced technologies and tools available, there are great opportunities for mapping correlations between genotypes and phenotypes and gaining further insight into our understanding of *Arthrospira*.

**Figure 1:**
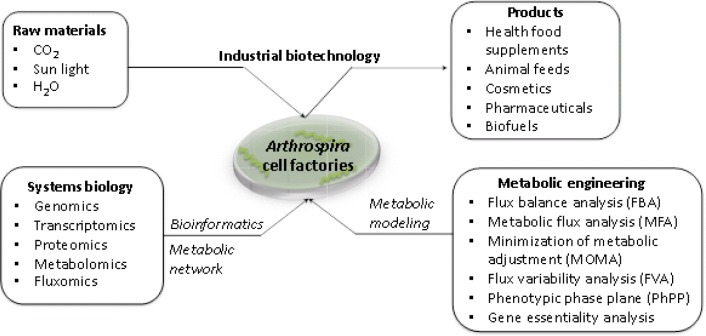
An integration of systems biology with metabolic engineering for advancing industrial biotechnology in order to develop efficient cell factories of *Arthrospira*.

To show an overview of systems biology and metabolic engineering through industrial biotechnology for the development of *Arthrospira* cell factories, we therefore illustrate the entire processes as shown in Figure 1. Initially, the process of developing *Arthrospira* cell factories from systems biology, i.e., multi-level omics, bioinformatics and metabolic networks, can be used to perform fundamental analysis of the cells. Afterwards, such *Arthrospira* cell factories are developed through metabolic engineering, i.e., mathematical modeling, and can be used to guide directed genetic manipulations. Finally, the developed cell factories of *Arthrospira* are used as workhorses for the industrial production process, i.e., conversion from raw materials to product formations.

In the following description, we firstly review how *Arthrospira* cell factories are mainly used for industrial applications. We then focus on the systems biology and the metabolic engineering toolboxes available for *Arthrospira*, i.e., genome sequencing projects on different *Arthrospira* strains for which the sequence data are publicly available, proteomics, genome-scale metabolic network and *in silico* modeling as well as their updated status and applications. Towards the end, we describe the perspective and future direction of *Arthrospira* cell factories.

## 2. Arthrospira as cell factories for industrial applications

*Arthrospira* (*Spirulina*) are filamentous non-N_2_-fixation cyanobacteria in a group of cyanobacteria which naturally grow in a high-salt alkaline open pond system in tropical areas [8–10] and utilize sunlight and CO_2_ to produce essential chemical compounds for life. The genus *Arthrospira* is comprised of approximately 51 strains within two common species, namely, *Arthrospira platensis* and *Arthrospira maxima*, that play an important role for industrial applications [11]. Table 1 summarizes the available information of various important biotechnological aspects of *Arthrospira* strains.

**Table 1:** Summary of different strains of *Arthrospira*'s potential substances for industrial biotechnology applications.

*Arthrospira* strains	Biotechnological importance		References

Potential products	Industries
*A. platensis* *A. maxima*	Food supplement	Dietary supplements	[10, 13, 15, 21, 25, 26, 61–63]
*A. platensis* *A. maxima*	Feed additive	Aquacultures and animal feeds	[64–66]
*Arthrospira*sp.	Vitamin B12	Pharmaceuticals	[19, 67, 68]
*A. platensis* *A. maxima*	Carotenoids	Pharmaceuticals	[22, 69–71]
*A. platensis* *A. maxima*	Tocopherols	Pharmaceuticals	[72, 73]
*A. maxima*	Phenolic acids	Pharmaceuticals	[74–76]
*A. platensis*	Selenium	Pharmaceuticals	[77–79]
*A. platensis*	Enzymatic antioxidants	Pharmaceuticals	[17]
*A. platensis* *A. maxima*	GLA	Pharmaceuticals	[22, 80]
*A. platensis*	Calcium spirulan	Pharmaceuticals	[22–24, 81, 82]
*A. platensis*	Polysaccharides	Pharmaceuticals	[82–84]
*A. platensis*	Phycocyanin	Pharmaceuticals Foods	[22, 85–88]
*A. maxima*	Biodiesel	Biofuels	[29, 89]
*A. platensis* *A. maxima*	H_2_	Biofuels	[30, 31, 90, 91]

*A. platensis* (*Spirulina platensis*) is widely used as a source for commercially-produced food supplements and animal feeds since the 1970s [12]. In addition, *A. platensis* has been consumed as a protein source for many years by North Africans and Mexicans [1]. As *A. platensis* contains high amounts of healthy nutritional molecules, such as beta-carotene, phycocyanin, vitamins, trace minerals, and polyunsaturated fatty acids [13–15], it is currently promoted as functional foods with safe consumption and widely sold in various health food stores in the forms of capsules, tablets, and powders. Recently, *A. platensis* has also played an important role for a wide range of antioxidant products, such as antioxidant enzymes (i.e., superoxide dismutase (SOD), catalase (CAT), peroxidase (PX), and ascorbate peroxidase (APX), as well as antioxidant compounds (i.e., carotenoids, tocopherols, vitamin C, and glutathione) which could be better used against cell death induced by free radicals than chemical antioxidants [16–18].

**Figure 2:**
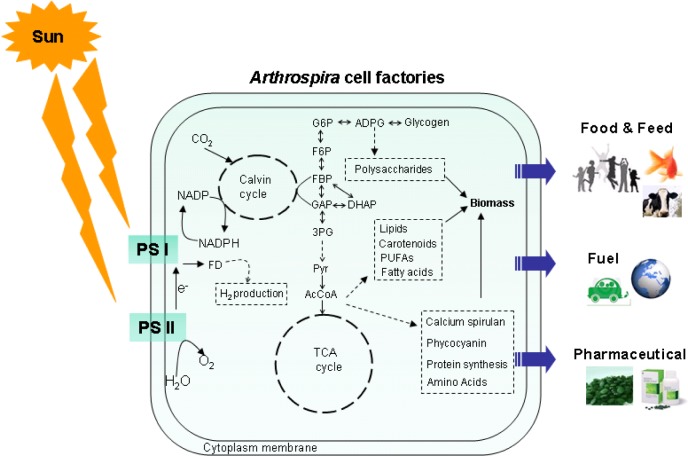
Schematic pathway diagram of the *Arthrospira* core metabolic process for the production of the chemical building blocks used as the precursors of high-value compounds discussed in this review. Abbreviations are: ADPG, ADP-glucose; G6P, glucose-6-phosphate; F6P, fructose-6-phosphate; FBP, fructose 1, 6-bisphosphate; GAP, glyceraldehyde phosphate; DHAP, dihydroxyacetone phosphate; 3PG, 1,3-bisphosphoglycerate; Pyr, pyruvate; AcCoA, acetyl-CoA; FD, hydrogenase-ferredoxin; PS I, photosystem I; PS II, photosystem II.

Moreover, there are many scientific articles which report the therapeutic benefits of this microorganism [19–22], such as invasive tumor inhibition [23], the inhibition of virus replication [24], and the inhibition of chronic disorders [25, 26] and cancers [27, 28]. Furthermore, *A. platensis* is potentially one microalgae that is capable of renewable energy production, which could decrease the effects of global warming. This is due to its potential to capture and convert CO_2_ and sunlight into various value-added products through cellular processes, e.g., photosynthesis, glycolysis, TCA cycle, and fatty acid and lipid biosynthesis. Hereby, *A. platensis* has become an attractive photobiological cell factory as presented in Figure 2.

Among the different species of microalgae [29], *A. platensis*, as well as *A. maxima*, were identified as being capable of growing in outdoor environments at a high rate showing high total lipids and biodiesel yields (9.2% and 7.5%, respectively). In particular, *A. maxima* strain CS-328 exhibited the high growth rate and showed the hydrogen yield by auto-fermentation among other cyanobacteria (up to 18% of hydrogen in the headspace) [30]. A noticeable example reported by Ananyev GM et al. showed that enhanced hydrogen production with 11-fold of hydrogen yield and 3.4-fold of hydrogen production rate from *A. maxima* was done by manipulating the equilibrium and thermodynamics of the hydrogenase reaction and removing the excreted products [31]. As described, these benefits correspond with other relevant nutritional value achievements from *Arthrospira*, showing the favorable industrial applications of cell factories.

In recent years, many efforts have been attempted to increase the biomass productivity of *Arthrospira* factories in various photobioreactors, such as in open raceway ponds with the biomass productivity of 28-46 mg L ^-1^day^-1^ [32] and in various types of closed photobioreactors with the biomass productivity of 42.3 mg L ^-1^day^-1^ [33], 135 mg L ^-1^day^-1^[34], and 220 mg L ^-1^day^-1^ [35]. Furthermore, several strategies for the improvement of *Arthrospira* biomass have been studied and proposed including changing physical parameters, i.e., inoculum ages [36], temperatures and pH [37], and solar UV radiations [38].Besides, biomass productivity and yields have been further enhanced in *Arthrospira* cultivations during the past decade by changing some of the nutrients from those in the conventional mediums to other carbon and/or nitrogen sources [39, 40], such as urea, ammonium andammonium chloride as nitrogen sources [41], as well as glucose and acetate as additional carbon sources in the photoheterotrophic culture of *Arthrospira* [42, 43]. However, these alterations of nutrients are still somewhat unproductive and impractical to be used in the cultivation of *Arthrospira* at a commercial scale due to the high costs of raw materials. Systems biology and metabolic engineering have therefore become increasingly significant to help identify new rational ways of enhancing the biomass biosynthesis rate in *Arthrospira*.

## 3. Systems biology and metabolic engineering toolboxes for *Arthrospira*

The cellular machinery complexity is always a biological challenge. The complex regulatory circuits occurring at different levels of cellular control within the cells are often a bottleneck for understanding living cells. To overcome this, the systems biology and metabolic engineering toolboxes are required. Here, we discuss these toolboxes through global information from genomics and proteomics as well as genome-scale metabolic models available for *Arthrospira*. At the end, we describe the status of existing metabolic models for *Arthrospira* and their applications.

### 3.1 Genomics

A recent publication in 2008 of the first genome sequence of *A. maxima* strain CS-328 [44] was released by the DOE-Joint Genome Institute through shotgun sequencing technologies (http://img.jgi.doe.gov/cgi-bin/w/main.cgi), the field of cyanobacterial genomics that has been of growing importance to biological studies. In the consequent time, there were two genome sequences of *Arthrospira* available, namely, *Arthrospira* sp. strain PCC 8005 and *A. platensis* strain Paraca, which contained about 100 contigs [44] and 1,000 contigs (http://img.jgi.doe.gov/cgi-bin/w/main.cgi), respectively. So far, these current genome sequences are still incomplete and under functional annotation with the overall aim to better understand the physiology and metabolic potential of *Arthrospira*. Considering further genomic evolution events (e.g. gene loss or gene transfer), this remains a big challenge [45] for the cyanobacterial field.

A few years later, there was an effort by the National Institute of Technology and Evaluation (NITE) for a complete genome sequencing project. In 2010, *A. platensis* strain NIES-39 was successfully released with a complete form of a single circular genome [46]. This has absolutely unlocked the hidden code of cyanobacterial life.

For fundamental research, *A. platensis* strain NIES-39 can be used as a reference genome for mapping, ordering and orientation of various contigs existing in the incomplete *Arthrospira* genomes. Moreover, it can be used for advancing comparative genomics studies, such as a comparative analysis of *A. platensis* between genomes of NIES-39 and C1 (PCC9438) [47]. *A. platensis* strain C1 (PCC9438) is a new genome recently published in 2012 by Cheevadhanarak et al. from King Mongkut's University of Technology Thonburi, Thailand [47]. Up to date, the comparative studies provide a survey in the basic knowledge of *Arthrospira* strains and their metabolic diversity which yields valuable insight into *Arthrospira* metabolism.

Regarding all possible characteristics of *Arthrospira* genomes available as listed in Table 2, the typical genome sizes are in between 5.0-6.7 Megabases (Mb) with an average GC content of 44.3-44.8%. In terms of gene prediction and functional annotation using bioinformatics, *Arthrospira* genome sequences include 5,370-6,630 protein-encoding genes and 30-45 RNA genes. Among these, 800-1,400 protein-encoding genes correspond with the KEGG metabolic pathway (Table 2). For the other functional enrichment analysis, interestingly the *Arthrospira* species have abundant functions involved in defense mechanisms (e.g. restriction modification, group II intron, CRISPR, and insertion elements (ISs) [44, 46, 47]. Through the genomic description as mentioned above, it suggests that *Arthrospira* species are feasibly versatile cell factories at a genetic level. Table 2 shows the status of genomics information available for the *Arthrospira* genomes.

**Table 2:** Summary of genomics information of *Arthrospira* strains.

Strains	Genome sizes (Mb)	Genes	No. of contigs	No. of scaffolds	NCBI Project IDs
*A. maxima* CS-328	6.00	5,73	129	129	29085
*A. platensis* Paraca	4.99	5,401	1,82	1,82	34793
*Arthrospira* sp. PCC 8005	6.14	5,718	119	16	40633
*A. platensis* NIES-39	6.78	6,676	18	1	42161
*A. platensis* C1	6.08	6,153	63	1	67617

### 3.2 Proteomics

Proteomics is one of highly-studied omics technologies for *Arthrospira* in order to achieve quantitative description of protein expression and its changes under the influence of biological perturbations. Currently, there are several proteomics studies of *Arthrospira* on the cellular response to the temperature stress. For instance, an interesting story by Hongsthong et al. [48] presented differential expression studies of *A. platensis* proteins during cold-induced stress. Their results showed that the up-regulated proteins in every subcellular fraction were involved in two-component response systems, DNA repairs, molecular chaperones, stress-induced proteins and proteins related to other biological processes (e.g. secretion system and nitrogen assimilation). A following study by Hongsthong et al. about the changes in protein expression of *A. platensis* was then examined upon exposure to high temperature [49]. Surprisingly, it revealed a number of proteins which were tolerant to high temperature stress and showed a relationship between temperature stress as well as nitrogen and ammonia assimilation [49]. Considering different conditions under low- and high-temperature stresses, Kurdrid et al. further showed that low-temperature stress was tightly linked to oxidative stress and photosynthesis; temperature stress was also suggested to be connected with nitrogen and ammonia assimilation. However, there was no specific mechanism for high-temperature stress response [50].

### 3.3 Genome-scale metabolic model

**Figure 3:**
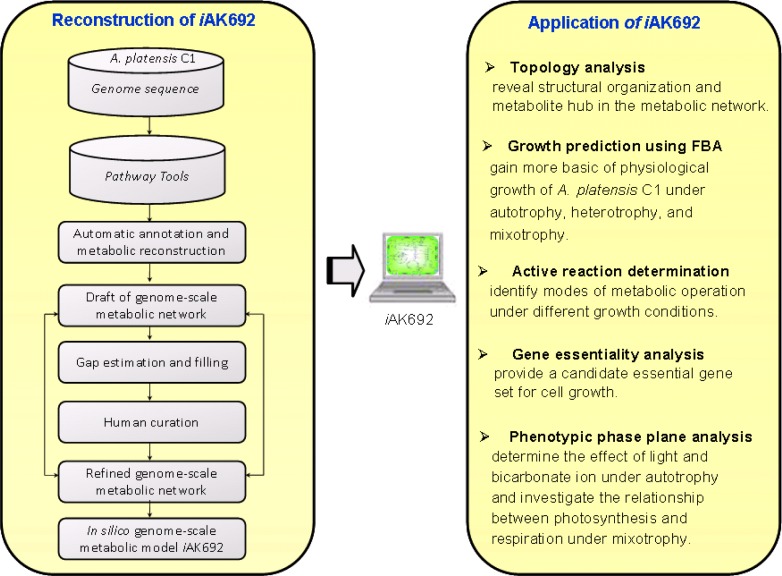
A schematic diagram of the development and applications of the first genome-scale model of *Arthrospira*, *i*AK692.

The availability of recent genomic sequences and annotations of *A. platensis* strain C1 [47] has made it possible to reconstruct its first genome-scale metabolic network so-called *i*AK692. This first comprehensive reconstruction of *Arthrospira* metabolism was proposed by the group of Klanchui et al. [51]. The genome-scale model was named *i*AK692 where AK is the name of the creator, Amornpan Klanchui, and 692 is the number of open reading frames (ORFs) included in the model. The schematic diagram of the model development and its applications is schematically illustrated in Figure 3. In brief, the metabolic network reconstruction of *A. platensis* strain C1 was initially started with automated construction using Pathway Tools software [52] to generate a draft metabolic network. This automated construction facilitates the top-down reconstruction approach which rapidly gives an overview network and visualization. Certainly, the quality of the large-scale reconstructed network depends on the quality of the initial annotated data. Hence, manual annotation was subsequently performed to increase the accuracy of the draft auto-generated network. Nonetheless, there are still a number of incomplete pathways (i.e. missing genes and reactions) indicating disconnections in the network. Missing reactions (referred to gaps) that resulted in dead-end metabolites and prevented the model simulation of cell growth were then identified. Therefore, an additional step for metabolic network refinement of *A. platensis* strain C1 was performed based on present information about cyanobacteria from biochemical literature, biochemistry textbooks, and online biochemical databases. In order to complete the pathways where no gene could be found in the metabolic reconstruction, the Blast algorithms and the advanced tools for the protein domain prediction against Pfam database were further used to determine the enzymatic gene functions of *A. platensis* strain C1. Towards the end, the refined metabolic network of *A. platensi*s strain C1 contained 692 metabolic genes, 658 metabolites, and 688 biochemical reactions [51]. Then, a stoichiometric model for flux balance analysis (FBA) modeling was formulated to investigate and predict the cellular physiology of *Arthrospira*. The stoichiometric model was employed for studying the metabolic states of *A. platensis* strain C1 under autotrophic, heterotrophic, and mixotrophic growth conditions. Interestingly, *i*AK692 model-based FBA was shown to correspond with the fermentation experiments during autotrophy, heterotrophy, and mixotrophy. In addition to the growth studies, the topological network analysis was then performed in order to reveal metabolic network properties of the *i*AK692 model. Through this topological analysis, the model could allow the large-scale *in silico* gene deletion and reaction activity analysis for a specific growth condition. In addition, *i*AK692 was employed to investigate the relationship between photosynthesis and respiration during *Arthrospira* growth by using phenotype phase plane analysis (PhPP). Clearly, the *i*AK692 model is not only used for global understanding of physiological growth behaviors and product formulations, but also as a platform for systems biology investigation leading to metabolic engineering and strain improvement [53] towards a diverse range of biotechnological abilities of *Arthrospira*.

Genome-scale metabolic models provide an additional framework for direct integration and analysis with high throughput-omics data and bridge the gap between knowledge of observed experiment and global metabolic network structure [54, 55]. These advantages of genome-scale metabolic models therefore allow us to investigate all possible behaviors of the cellular systems responding to environmental changes. Certainly, a new strategy for strain improvement and biological process development for *Arthrospira* can be further identified.

**Figure 4:**
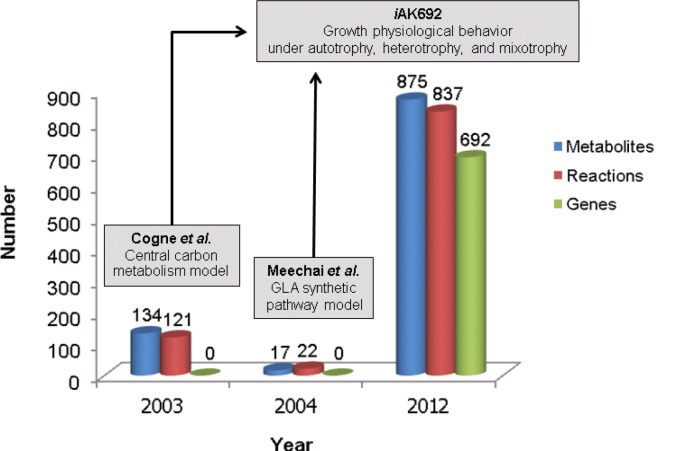
Development of metabolic models of *A. platensis*. Each box represents a summary of a name and scope of a metabolic model. The number of metabolites (blue bar), reactions (red bar), and genes (green bar) are shown.

In addition to the recent *i*AK692 model, there were different versions of smaller-scale metabolic models earlier reconstructed for *Arthrospira* as illustrated in Figure 4. Halfway through 2003, Cogne et al. [56] constructed the first small-scale metabolic network of *Arthrospira* based only on data collection from literature and experimental data comprising biological pathways and cell physiology evidences. Cogne's metabolic network contained 121 reactions and 134 metabolites which were distributed into central carbon metabolism (i.e. glycolysis, pentose phosphate pathway and TCA cycle) and abundant anabolism reactions leading to biomass constitution, exopolysaccharide (EPS) production, and energy aspects. Once Cogne's metabolic network was converted into a stoichiometric model, metabolic flux analysis was performed for quantitative analysis of growth under autotrophy. Interestingly, Cogne et al. found that the metabolic model revealed metabolic constraints with respect to NADH and H^+^ balances leading to the maximal yield of the *Arthrospira* growth-associated EPS.

Shortly after the Cogne et al. model, another version of a small-scale metabolic network of *Arthrospira* was reconstructed by Meechai et al.,[57] with the overall purpose aimed at a GLA synthesis pathway. Meechai's metabolic network reconstruction was initially carried out with only literature and experimental data. It contained 22 reactions and 17 metabolites [57] which were involved in the central carbon metabolism, the anaplerotic pathway, the photosynthesis, the GLA synthesis pathway and the biomass synthesis pathway. After Meechai's stoichiometric model was formulated, the metabolic flux analysis was performed for overall metabolic distribution studies in the GLA synthesis pathway between wild-type and mutant strains. Once the model was validated with the physiological data, wild-type BP and a high-GLA producing mutant strain Z19/2 were experimentally studied on the GLA synthesis pathway. Meechai's model suggests that a metabolic reaction converting acetyl-CoA into malonyl-CoA is a bottleneck for GLA synthesis of *Arthrospira*. Moreover, the model suggests the addition of simulation factors, such as NADPH and MgCl_2,_ which resulted in higher GLA production rates in the wild-type strain.

## 4. Perspective and future direction on *Arthrospira* cell factories

Needless to say, *Arthrospira* are attractive cyanobacteria to be used as efficient cell factories for the production of many valuable compounds useful for food, feed, fuel and pharmaceutical industries. This presents a grand challenge for the researchers to realize the enormous biotechnological potential of *Arthrospira*. Unfortunately, a stable and reliable system for efficiently transferring exogenous genes into *Arthrospira* is not yet available, thus the use of *Arthrospira* as cell factories is still limited. Nevertheless, much progress has been made in developing systems for gene manipulation in the *Arthrospira* species. For instance, a system for gene manipulation by certain restriction endonuclease enzymes were identified and characterized in *A. platensis* [58, 59]. In addition, a transformation using a natural Tn5 transposase-transpon-cation liposome complex with electroporation that efficiently transferred chloramphenicol acetyltransferase gene to *A. platensis* strain C1 (PCC9438) has been reported [60]. The limited successful establishment of the genetic transformation system is mainly attributed to the defense mechanism systems found in *Arthrospira* such as restriction and modification enzymes (RM) as well as CRISPR [47]. Hence, the developing area of the strategies and genetic tools for manipulation of these cyanobacteria are crucial for further success of genetic engineering efforts.

The increasing availability of whole genome sequences and the present knowledge relating to *Arthrospira*'s metabolic and regulatory systems allow the researchers to gain insights into this organism at the systematic levels. This knowledge can now be used to address biological meanings and answers through possible biological questions, such as how the transformation systems in *Arthrospira* work in terms of function and which alternative ways can be used to establish novel genetic tools for genetic manipulation of *Arthrospira*. For example, thorough analysis of the *Arthrospira* genome sequence would provide an in-depth understanding of its unique RM systems, thereby enabling the development of efficient transformation strategies. For instance, with proper design and modification of an exogenous gene, the RM defense mechanism can be avoided, and thus a stable transformation can be achieved. Additionally, the recently published genome-scale metabolic model of *A. platensis* has also been used as a systems biology platform for analyzing *A. platensis* high-throughput data at both transcriptomics and proteomics levels within the metabolic context. In the future, once the bioinformatics algorithms, the mathematical models and the *Arthrospira* phenotypic characterization of different mutants suited for metabolic engineering have advanced, new findings by the genome-scale model shall certainly pave a way for comprehensive studies of cellular systems, as well as the development of desirable *Arthrospira* cell factories for various industrial applications. Beyond this, we look forward to expand and develop the biotechnological potential of *Arthrospira* as biological factories for applied use in a greener era.
